# Prognostic value of endotoxin activity assay in patients with severe sepsis after cardiac surgery

**DOI:** 10.1186/1476-9255-10-8

**Published:** 2013-03-06

**Authors:** Michail Yaroustovsky, Marina Plyushch, Dmitry Popov, Natalia Samsonova, Marina Abramyan, Zakhar Popok, Nickolay Krotenko

**Affiliations:** 1 Bourakovsky Institute of Cardiac Surgery, Bakoulev Scientific Centre for Cardiovascular Surgery, Moscow, Russia

**Keywords:** Sepsis, Endotoxin, Endotoxin activity assay, Cardiac surgery

## Abstract

**Background:**

To evaluate the prognostic value of endotoxin activity assay (EAA) in adult patients with suspected or proven severe sepsis after cardiac surgery

**Methods:**

Blood samples taken from 81 patients immediately after the diagnosis of severe sepsis were tested with the EAA. Patients were divided into 3 groups: low (<0.4, n = 20), moderate (0.4-0.59, n = 35) and high (≥0.6, n = 26) EAA levels.

**Results:**

Gram-negative bacteraemia was found in 19/55 (35%) of cases with ЕАА <0.6 and in 11/26 (42%) of cases with higher ЕАА, p = 0.67. Mortality at 28 days in Groups 1, 2 and 3 was 20%, 43% and 54%, respectively. Patients with an EAA higher than 0.65 had a higher 28-day mortality than those with lower EAA values (18/26 – 69% vs. 19/55 – 34.5%; p = 0.0072). ROC analysis for the prediction of 28-day mortality revealed an AUC for APACHE II scores, EAA and PCT of 0.81, 0.73 and 0.66, respectively.

**Conclusions:**

EАА might be useful for recognising patients who have an increased risk of mortality due to severe sepsis.

## Background

The role of endotoxin in the pathogenesis of sepsis is well-known [1]. Endotoxin activates the release of cytokines and other biologically active components. Developing of systemic inflammatory response syndrome leads to disturbances in membrane permeability together with multiple organ dysfunction syndrome and, often, death. Therefore, clinicians require a precise laboratory diagnostic tool for endotoxin detection for the timely initiation of specific treatment.

Quantitative techniques for the measurement of endotoxin levels in blood have been known for more than 40 years [2] and are based on the application of the LAL endotoxin assay (Limulus Amebocyte Lysate). Recently, a new method for assessing endotoxin concentration in blood has been developed – the Endotoxin Activity Assay (EAA) [3,4]. Experimental observations [4] revealed that the results of EAA testing correlate with the level of endotoxemia, as determined by the LAL method: At EAA = 0.4, the endotoxin level is comparatively low, approximately 25–50 pg/mL, but with an increase of EAA to 0.6, the endotoxin level rises to 100–200 pg/mL.

EAA might be helpful in the early recognition of patients who are at high risk of developing severe sepsis and septic shock. EAA levels lower than 0.4 represent a low risk for the development of severe sepsis and in most cases, confirm the absence of a severe gram-negative infection. EAA values within the range of 0.4-0.59 are considered average and correspond to an increased risk of severe sepsis. EAA levels of 0.6 and greater represent a high risk of mortality and development of severe sepsis and septic shock development. These data are reflected in the integrated acute physiology and chronic health evaluation score (APACHE II): a low EAA level corresponded to a mortality of 10.9% and an APACHE II score of 13.3, whereas an average and high EAA were associated with a mortality of 13.2% and 16.8%, respectively, and APACHE II scores of 15.3 and 17.6, respectively [4]. A prospective study conducted by D. Klein et al. on 53 patients with septic shock revealed a mortality of 16% at EAA levels lower than 0.4 and 34% with EAA levels between 0.4 and 0.59. EAA levels greater than 0.6 corresponded to a mortality increase of 50% and greater [5,6]. Moreover, EAA testing provides an opportunity to estimate the effectiveness of treatment.

Contemporary clinical practice entails the complex management of sepsis, including haemodynamic and respiratory support, prevention of thromboembolism and gastrointestinal bleeding, nutritional support, and therapy with steroids [7]. In addition, detoxification therapy, together with extracorporeal blood purification based on the application of selective LPS-sorbents, seems to be useful [8,9].

Laboratory indicators of endotoxemia together with clinical findings and data from other diagnostic methods serve as criteria for the assessment of sepsis severity, treatment effectiveness and outcomes. Investigations that estimate the prognostic value of the EAA method are currently lacking. So, aim of the study was to evaluate the prognostic value of EAA in adult patients with suspected or proven severe sepsis after cardiac surgery.

## Methods

The study was approved by the Ethics Committee of Bakoulev Scientific Centre for Cardiovascular Surgery. Between November 2009 and February 2012, 3245 adult patients underwent open-heart surgery in our Institute. In a single-centre prospective observational study were included 81/3245 (2.5%) patients with suspected or proven severe sepsis. Patients’ initial characteristics are shown in Table 1.

**Table 1:** Patient characteristics

**Parameters**		**Overall population, n = 81**	**Survivors, n = 44**	**Non-survivors, n = 37**	p-value*
Age, years		56 (47.5-64.5)	53 (42–61)	59 (52–66)	0.06
Male sex		45/81 (56%)	23/44 (52%)	22/37 (59%)	0.67
APACHE II scores		20 (10–30)	11 (8–26)	28 (15–31)	0.0008
Renal failure		41/81 (50.6%)	20/44 (45.5%)	24/37 (64.9%)	0.19
Dialysis requirement		19/81 (23.5%)	6/44 (13.6%)	13/37 (36.4%)	0.44
Epinephrine	Receivers	74/81 (91.4%)	40/44 (90.9%)	34/37 (91.9%)	0.81
	Dose, mcg/kg/min	0.08 (0.05-0.1)	0.07 (0.05-0.1)	0.09 (0.06-0.1)	0.31
Dopamine	Receivers	53/81(65.4%)	29/44 (65.9%)	24/37 (64.9%)	0.89
	Dose, mcg/kg/min	5 (4–7)	5.5 (5–8)	5 (3–6)	0.06
Dobutamine	Receivers	26/81(32%)	14/44(31.8%)	11/37(29.7%)	0.97
	Dose, mcg/kg/min	5.75 (4–8)	5.15 (4.25-7)	6 (4–8)	0.64
Norepinephrine	Receivers	12/81(14.8%)	5/44(11.4%)	9/37(24.3%)	0.02
	Dose, mcg/kg/min	0.07 (0.05-0.1)	0.08 (0.05-0.15)	0.06 (0.05-0.09)	0.46
WBC, х10^9^/l		13.2 (10.5-19.3)	13 (10.6-20.8)	13.4 (10–18.9)	0.83
LAL,EU/mL		0.72 (0.36-0.72)	0.72(0.36-0.72)	0.72 (0.36-1.08)	0.65
РСТ, ng/mL		5.07 (1.25-13.72)	3.06 (0.72-8)	5.69 (3.19-23.34)	0.05
ЕАА		0.49 (0.39-0.66)	0.45 (0.33-0.58)	0.6 (0.45-0.69)	0.009

* between survivors and non-survivors.

The average age was 56 (47.5-64.5) years. Forty-five of the patients underwent valvular surgery, whereas 12 underwent combined myocardial revascularisation, 19 underwent isolated surgery for coronary anomaly correction, 4 underwent surgery for hypertrophic cardiomyopathy accompanied by correction of left ventricular outflow tract obstructions, and in 1 patient with dilated cardiomyopathy, an orthotopic heart transplantation was performed.

The early postoperative period in all studied patients was complicated by low cardiac output syndrome (LVEF 15-28%), which required inotropic support by two or more sympathomimetic agents. In 10 cases, an intra-aortic balloon pump was applied. The presence of respiratory failure with poor gas exchange demanded prolonged mechanical lung ventilation. Patients developed severe sepsis on ICU day 7 on average (range: day 4–8), defined as the development of systemic inflammatory response syndrome (SIRS) combined with a suspected or proven locus of bacterial infection alongside multiorgan dysfunction. The most common source was ventilator-associated pneumonia, found in 66/81 (81%) patients and confirmed by clinical, X-ray and laboratory data.

All patients received treatment for severe sepsis according to approved guidelines [7], including antimicrobial therapy in a de-escalation regimen adjusted by bacteriological data.

After the onset of severe sepsis, clinical and laboratory parameters were recorded as well as blood samples for procalcitonin (PCT) and endotoxin assays were taken. Endotoxin levels were assessed by means of the LAL test (Biowhitakker, USA) and by EAA, which was carried out by the luminol chemiluminescence method (Spectral Diagnostics Inc, Canada). EAA is based on the principle that endotoxin binds to antiendotoxin antibodies (IgM) and is then delivered to neutrophils via complement receptors. In the presence of zymosan and luminol, neutrophils undergo a respiratory burst accompanied by light emission. The light produced is quantified by a chemiluminometer, and its intensity is proportional to the amount of endotoxin.

PCT plasma concentrations were measured by the VIDAS B·R·A·H·M·S PCT test (bioMérieux, France).

To compare the predictive values of EAA, LAL-test, PCT, and APACHE II, receiver operating characteristic (ROC) curves were constructed, and the area under the curve (AUC) was determined. The outcome variable was 28-day mortality.

Statistical analysis was performed with SPSS software, version 20 (SPSS, Inc., Chicago, Ill). Discrete variables were expressed as counts (percentage), and continuous variables were expressed as a median and inter-quartile range. Statistical differences between groups were assessed with the Mann-Whitney U-test. Correlations were determined with Spearman's rank method (two-sided). P-values less than 0.05 were considered statistically significant.

## Results

Clinical and laboratory data from studied patients are shown in Table 1.

There were no significant differences between survivors and non-survivors in terms of age, gender, rate of renal failure, dialysis requirement, need for inotropic support (except norepinephrine) and WBC count. APACHE II scores were significantly higher in non-survivors. In contrast to PCT and LAL-test results, levels of EAA were significantly greater in deceased patients.

Forty-four of 81 studied patients (54%) had positive bronchoalveolar lavage (BAL) cultures for various bacterial species: Acinetobacter baumannii in 19 patients; Klebsiella pneumoniae in 11 patients; Pseudomonas aeruginosa in 7 patients; and coagulase-negative Staphylococci in 7 patients. The same bacteria were responsible for positive blood cultures, which were observed in 30 patients (37%): Klebsiella pneumoniae in 2 patients; Acinetobacter baumannii in 10 patients; Pseudomonas aeruginosa in 5 patients; and coagulase-negative Staphylococci in 13 patients. The rate and spectra of positive BAL and blood cultures were similar between survivors and non-survivors.

Patients were divided into 3 groups by EAA results: EAA <0.4 (Group I, low EAA, n = 20); EAA between 0.4 and 0.59 (Group II, elevated EAA, n = 35); and EAA ≥ 0.6 (Group III, high EAA, n = 26). Respective group data are shown in Table 2.

**Table 2:** Clinical and laboratory indices

** Group**	**ЕАА**	**LAL,EU/mL**	**РСТ, ng/mL**	**APACHE II**	**28-day mortality,%**
Group I, n = 20	0.3 (0.26-0.34)	0.36 (0.36-0.72)	3.49 (1.25-8.75)	10 (6–13)	20
Group II, n = 35	0.48* (0.44-0.52)	0.72 (0.36-0.72)	3.68 (0.95-7.7)	15 (9–20.5)	43
Group III, n = 26	0.69** (0.65-0.73)	0.72** (0.7-1.44)	9.3** (4.2-28.3)	30,5** (28–32)	54**

* p _I-II_ <0.05.

** p _I-III_ <0.05.

Gram-negative bacteraemia was found in 19/55 (35%) of cases with ЕАА less than 0.6 and in 11/26 (42%) of cases with higher ЕАА (p = 0.67).

We obtained higher levels of blood plasma endotoxin, measured by the LAL-test, as well as greater PCT concentrations, APACHE II scores and mortality in patients with moderate and high EAA levels compared to patients with low EAA levels. These differences were statistically significant.

There was a moderate correlation of ЕАА with LAL-test results and РСТ (r = 0.53, p = 0.002 and r = 0.27, p = 0.03 respectively). We did not find a correlation between LAL test results and РСТ levels (r = 0.17, p = NS).

Also APACHE II scores correlated with EAA and PCT (r = 0.33, p = 0.005 and r = 0.31, p = 0.01 respectively), but not with LAL-test (r = 0.29, p = NS).

**Figure 1:**
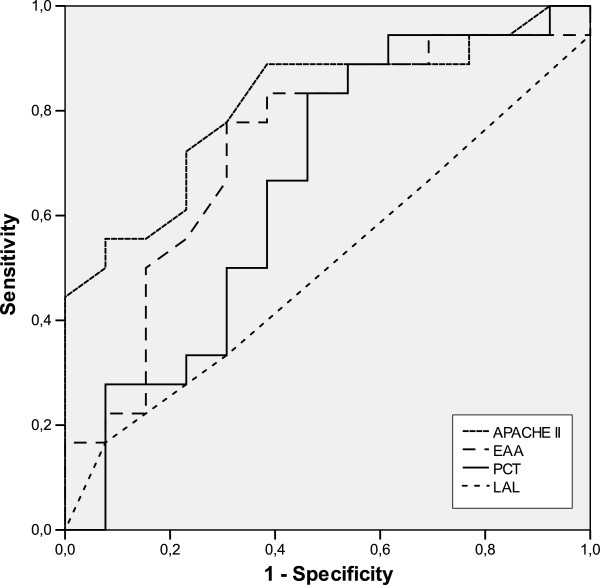
Receiver operating characteristic curve of serum levels of EAA, LAL, PCT and APACHE II in predicting 28-day mortality.

ROC analysis comparing the accuracy for the prediction of 28-day mortality revealed areas under the receiver operating characteristics curve (AUC) for APACHE II scores, EAA and PCT of 0.81, 0.73 and 0.66, respectively (Figure 1). The optimal prognostic cut-offs (maximum combined sensitivity and specificity) related to 28-day mortality with corresponding values are listed in Table 3.

**Table 3:** ROC analysis data

**Parameter**	**AUC (95% CI)**	**Optimal cut-off value**	**Se,%**	**Sp,%**
APACHE II	0.81 (0.66-0.96)	11.5	78	69
EAA	0.73 (0.55-0.92)	0.65	72	69
PCT	0.66 (0.45-0.87)	4.76	67	62

*Se* sensitivity, *Sp* specificity, *AUC* area under the curve.

The AUC for EAA (0.73) was higher than the AUC for PCT and lower that of APACHE II (0.66 and 0.81, respectively). AUC for LAL was 0.5 (0.3-0.71), so we did not calculate further characteristics for this marker.

Patients with EAA results higher than 0.65 had a 28-day mortality that was significantly higher than those with a lower EAA value (18/26 – 69% vs. 19/55 – 34.5%; p = 0.0072).

## Discussion and conclusions

Sepsis, severe sepsis and septic shock still represent obstacles in modern medicine due to a high morbidity and unacceptable mortality. Therefore, it is important to introduce new diagnostic tools and therapeutic interventions for the early recognition and treatment of patients in such severe conditions.

It is well-known that the clinical signs and symptoms of systemic inflammatory response syndrome (SIRS) are non-specific, so the use of biomarkers such as PCT is required. This marker, triggered mainly by LPS, IL-6 and TNF-α, reflects the response of the macroorganism to a bacterial load and can be used as an early, sensitive and specific diagnostic tool, as well as to guide antibiotic treatment [10].

Endotoxemia is a common finding in critically ill patients and can originate either from a focal gram-negative infection or the gastro-intestinal tract due to dysfunction of its barrier [11]. In our study, gram-negative bacteraemia was observed in 19/55 (35%) of patients with an ЕАА < 0.6 and only in 11/26 (42%) of cases with an ЕАА ≥ 0.6, which may reflect the load from other sources, mainly the gut [12].

Endotoxin exposure can induce systemic inflammation, progressing to sepsis that results in shock and multiorgan failure. Taking all of this into consideration, it is important to have the opportunity to measure the blood levels of LPS, which can guide therapy (e.g., LPS adsorption to prevent or attenuate the inflammatory response).

For many years, the detection of bacterial endotoxin was performed by the LAL-test. This method has some serious limitations; one of the most important is a poor specificity for LPS [13]. The endotoxin activity assay may provide an opportunity to perform a prompt and objective assessment of endotoxin concentration in a patient’s blood.

We found that EAA significantly correlates with LAL-test results (r = 0.53, p = 0.002) and with PCT (r = 0.27, p = 0.03). This may be explained by the fact that the immune response functions as a trigger, and the presence of LPS in the blood will not necessarily lead to activation of the inflammatory cascade and the development of systemic inflammation. That is why the application of methods to detect endotoxin, together with markers of the macroorganism response (e.g., PCT), yielded the best results.

Monti et al. published data on the interaction between ЕАА and prognosis in critically ill septic shock patients. According to that study, all patients with low endotoxin activity (<0.4) survived. Patients with intermediate (between 0.4 and 0.6) and high (≥0.6) EAA levels had a mortality of 17% and 37%, respectively [14]. The data obtained in the present study allow us to assume that endotoxin activity levels exceeding 0.65 indicate increased 28-day mortality and in the presence of other clinical signs of severe infection. AUCs for APACHE II and EAA as a predictors of 28-day mortality were higher, than for PCT which can be used as a criterion to initiate specific treatment, including earlier introduction of selective LPS adsorption into the treatment complex.

Early selective haemoperfusion with a polymyxin B cartridge (PMX) improves the results of complex intensive therapy for sepsis and multiorgan failure [9]. It was shown that EAA can be used as a criterion for the initiation of LPS adsorption procedures to decrease the 28-day mortality in patients with severe sepsis from 75% to 45.5% [15]. During this treatment, EAA levels decrease, which reflects the effectiveness of endotoxin removal from the blood of patients with sepsis. Other studies found a similar reduction in ICU mortality (from 45% to 16%, p = NS due to small sample size) and improvement in the clinical condition of septic shock patients who received adjuvant therapy with PMX [16]. A reduction in the endotoxin activity level by 33–80% was also demonstrated in the last European multicentre randomised controlled trial, “EUPHAS” [17].

Determining endotoxin activity by EAA allows the selection of patients at the highest risk of developing severe infections as well as predicting the outcome. This marker gives an early indication for specific intensive therapy for sepsis and facilitates the evaluation of its effectiveness.

## Abbreviations

APACHE II: Acute physiology and chronic health evaluation score; AUC: Area under the curve; EAA: Endotoxin activity assay; ICU: Intensive care unit; IL-6: Interleukin-6; LAL: Limulus Amebocyte Lysate; LPS: Lipopolysacharide; LVEF: Left ventricle ejection fraction; PCT: Procalcitonin; PMX: Polymyxin B cartridge; ROC: Receiver operating characteristic curves; SIRS: Systemic inflammatory response syndrome; TNF-alpha: Tumor necrosis factor alpha; WBC: White blood cells.

## Competing interests

The authors declare that they have no competing interests.

## Authors’ contributions

MY and MP contributed to the conception, design and methodology of the study, MP also performed laboratory analyses. DP and MA analyzed the results and wrote the manuscript. MY and NS reviewed the manuscript. ZP and NK collected samples and provided clinical data. All authors read and approved the final manuscript.
